# Sex Differences in the Association Between Obesity and Cognitive Impairment in a Low-Income Elderly Population in Rural China: A Population-Based Cross-Sectional Study

**DOI:** 10.3389/fneur.2021.669174

**Published:** 2021-07-09

**Authors:** Dandan Guo, Xin Zhang, Changqing Zhan, Qiuxing Lin, Jie Liu, Qiaoxia Yang, Jun Tu, Xianjia Ning, Jinghua Wang, Yijun Song

**Affiliations:** ^1^Department of Neurology, Tianjin Medical University General Hospital, Tianjin, China; ^2^Department of Neurology, Wuhu No.2 People's Hospital, Wuhu, China; ^3^Laboratory of Epidemiology, Tianjin Neurological Institute, Tianjin, China; ^4^Key Laboratory of Post-neuroinjury Neuro-Repair and Regeneration in Central Nervous System, Tianjin Neurological Institute, Ministry of Education, Tianjin, China; ^5^Department of Cardiology, Tianjin Medical University General Hospital, Tianjin, China; ^6^Department of General Medicine, Tianjin Medical University General Hospital, Tianjin, China

**Keywords:** cognitive impairment, obesity, body mass index, waist circumstance, sex difference

## Abstract

**Background:** Obesity is a potentially modifiable risk factor for cognitive impairment. However, sex-specific relationships between obesity and cognitive impairment in late life remain unclear.

**Objective:** We aimed to assess sex differences in the association between various obesity parameters and cognitive impairment in a low-income elderly population in rural China.

**Methods:** A population-based cross-sectional study was conducted to collect basic information from elderly residents aged 60 years and older from April 2014 to August 2014 in rural areas of Tianjin, China. Obesity parameters, including body mass index (BMI) and waist circumference (WC), and Mini Mental State Examination scores were measured, and the relationships between these variables were assessed.

**Results:** A total of 1,081 residents with a mean age of 67.70 years were enrolled in this study. After adjusting for age, educational attainment, smoking status, drinking status, physical exercise participation, and the presence of diabetes and hyperlipidemia, blood pressure group; a high BMI was found to be associated with an increased prevalence of cognitive impairment in elderly women. Each 1-unit increase in BMI was associated with a 5.9% increase in the prevalence of cognitive impairment. WC was related to the prevalence of cognitive impairment in elderly men, and each 1-cm increase in WC was associated with a 4.0% decrease in the prevalence of cognitive impairment. However, there were no significant associations between WC and cognitive function in women or between BMI and cognitive impairment in men.

**Conclusion:** A greater WC was positively associated with better cognitive function in low-income elderly men in rural China, whereas a higher BMI was associated with an increased risk of cognitive impairment in elderly women, independent of sociodemographic, lifestyle, and health-related comorbid factors. Our results suggest weight management of elderly women in rural China may have cognitive benefits. However, randomized controlled trials would be needed to confirm causality.

## Introduction

Cognitive disorders, including dementia and mild cognitive impairment, have become a global public health priority for aging populations (1, 2). According to the World Alzheimer Report 2019, there were over 50 million people living with dementia globally, and this number is estimated to increase to more than 152 million by 2050 (3). The current annual costs related to dementia worldwide were approximately 1 trillion USD in 2019, a figure set to double by 2030 (3). Moreover, the prevalence of dementia has been increasing rapidly over the past two decades in China, from 2.30 to 6.44%, especially among populations in rural areas with low educational attainment (4, 5). Meanwhile, the prevalence of obesity is also increasing worldwide and has become a global epidemic among older people (6). In China, the overall prevalence of general obesity among adults has increased 3-fold, from 3.3% in 2004 to 14.0% in 2014, posing a huge economic and social burden (7).

Obesity has often been recognized in the literature as a potentially modifiable risk factor for cognitive impairment (8, 9). The effects of obesity on cognitive function appear to vary by age and obesity status. Most of the recent studies have demonstrated that general obesity at midlife was related to an increased risk of cognitive decline in later life, especially for episodic memory and executive function (10–12). However, the association between obesity and cognitive impairment in the elderly is unclear. Several studies have demonstrated that general obesity as classified using body mass index (BMI) was associated with a decreased risk of cognitive impairment in the elderly, while abdominal obesity was harmful to cognitive function (13–15). In contrast, other studies have reported that both general and abdominal obesity were associated with an increased risk of cognitive dysfunction in old age (16, 17). Furthermore, few studies have reported the sex-related relationships between obesity and cognitive impairment, and these results were inconsistent (18–20).

The prevalence of cognitive impairment in rural China is notably higher than that in urban areas (5). However, the relationship between obesity in late life and cognitive impairment remains unclear among low-income populations in rural China. Additionally, sex differences in the associations between obesity and cognitive impairment are not well-known in China. Thus, the aim of this study was to assess the sex differences in the association between obesity and cognitive impairment among a low-income elderly population in rural China.

## Methods

### Study Population

This was a population-based, cross-sectional study conducted from April 2014 to August 2014 in rural areas of Tianjin, China. The participants were from the Tianjin Brain Study, which has been described previously (21, 22). All residents aged 60 years and older without vision and auditory dysfunction were invited to participate in this study. However, individuals with a history of myocardial infarction, stroke, congenital hypophrenia, and mental illness were excluded from participation.

The study was approved by the ethics committee of Tianjin Medical University General Hospital and conformed to the Declaration of Helsinki regarding the use of human subjects. Written informed consent was obtained from each patient during recruitment.

### Risk Factors and Physical Examinations

This study was conducted through face-to-face interviews by a trained research staff. A pre-designed questionnaire was used to collect the following information: demographic information (including name, sex, date of birth, and educational level), individual medical history (including the presence of hypertension, diabetes mellitus, and hyperlipidemia), and lifestyle factors (including smoking, drinking, and physical exercise). A physical examination was performed to obtain information on height, weight, and waist circumference (WC) with subjects wearing thin clothes. BMI was calculated as the individual's weight (kg) divided by the square of the individual's height (m^2^). Blood pressure (BP) measurements were performed using an electronic sphygmomanometer. Subjects were asked to remain resting in a sitting position for 15 min before testing; BP was measured three times, and the mean was obtained. The levels of fasting blood glucose (FBG), total cholesterol (TC), triglycerides (TG), high-density lipoprotein cholesterol (HDL-C), and low-density lipoprotein cholesterol (LDL-C) were tested in the central laboratory of Tianjin Ji County People's Hospital. Both the Mini Mental State Examination (MMSE) and Montreal Cognitive Assessment (MoCA) scales were used for screening cognitive impairment owing to their high sensitivity and specificity (23). However, considering that the content of the MoCA questionnaire was not suitable for elderly with low education in rural China, the MMSE questionnaire was used to assess cognitive impairment.

### Cognitive Impairment Criteria

The diagnostic criteria for cognitive impairment were based on MMSE scores and educational levels. Cognitive impairment was defined as an MMSE score <17 points in the group with no formal schooling, <22 points in the group with a primary school education, and <26 points in the group with a junior school education and above (24).

### Definitions

Hypertension was defined as a systolic blood pressure (SBP) ≥ 140 mm Hg, diastolic blood pressure (DBP) ≥ 90 mmHg, the use of antihypertensive drugs, or a history of hypertension. Stage I hypertension was defined as SBP ≥ 140 mm Hg and < 160 mm Hg or DBP ≥ 90 mm Hg and < 100 mm Hg, stage II hypertension was defined as SBP ≥ 160 mm Hg and < 180 mm Hg or DBP ≥ 100 mm Hg and < 110 mm Hg, and stage III hypertension was defined as SBP ≥ 180 mm Hg or DBP ≥ 110 mm Hg. Diabetes was defined as FBG ≥ 7.0 mmol/L, the use of diabetes medication, or a self-reported history of diabetes. Hyperlipidemia was defined as total cholesterol ≥5.18 mmol/L, low-density lipoprotein cholesterol ≥3.37 mmol/L, triglycerides ≥ 1.70 mmol/L, or high-density lipoprotein cholesterol < 1.04 mmol/L, or with diagnosis of hyperlipidemia. General obesity was defined as a BMI ≥ 28.0 kg/m^2^, and overweight was defined as a BMI ≥ 24.0 kg/m^2^ and < 28.0 kg/m^2^. Central obesity was defined as a WC >90 cm for men and >80 cm for women. Besides, BMI and WC were categorized into sex-specific quartiles. For men, first quartile of BMI was defined as <22.23 kg/m^2^; second quartile was 22.23–24.45 kg/m^2^; third quartile was 24.46–26.69 kg/m^2^ and fourth quartile was ≥ 26.70 kg/m^2^. First quartile of WC in men was defined as < 82 cm; second quartile was 82–87 cm; third quartile was 88–95 cm and fourth quartile was ≥ 96 cm. For women, first quartile of BMI was defined as <22.85 kg/m^2^; second quartile was 22.85–25.10 kg/m^2^; third quartile was 25.11–27.58 kg/m^2^ and fourth quartile was ≥ 27.59 kg/m^2^. First quartile of WC in women was defined as < 84 cm; second quartile was 84–88 cm; third quartile was 89–94 cm and fourth quartile was ≥95 cm. Smoking was defined as smoking ≥ 1 cigarette daily for more than 1 year. Drinking was defined as drinking >50 mL of alcohol at least once per week for more than 6 months.

### Statistical Analysis

Continuous variables are summarized as means and standard deviations; Student's *t-*test was used to compare the differences between the two groups. Categorical variables are summarized as numbers with frequencies; the chi-square test was performed to compare the differences between the two groups. The participants were categorized into four age groups (60–64, 65–69, 70–74, and ≥75 years), three educational groups according to the length of formal education (0–5, 6–8, and ≥9 years), and four BP groups (normal, stage I hypertension, stage II hypertension, and stage III hypertension). Multiple logistic regression analyses were used to evaluate the relationship between BMI or WC (continuous variables) and cognitive impairment by sex. Independent variables in the multivariate analyses were those factors found to be significant in the univariate analyses. Box-Tidwell method (25) was used to test linear relationship between continuous independent variables (BMI/WC) and dependent variable logit conversion values (all *P* > 0.05). Thus, BMI and WC were considered as continuous variables in multiple logistic regression analyses. Moreover, BMI and WC were also evaluated as categorical variables in multiple logistic regression analyses, divided by sex-specific quartiles. BMI and WC were included in different equations separately because of their high collinearity.

The multivariate analysis results are presented as adjusted odds ratios (ORs) and 95% confidence intervals (CIs) after adjusting for covariates. Additionally, three logistic regression models were developed to examine the associations of BMI and WC with cognitive impairment by sex. Model 1 involved adjusting for age and educational level (categorical variables). Model 2 was based on Model 1 and additionally adjusted for lifestyle factors (smoking, drinking, and physical exercise). Model 3 was based on Model 2 and additionally adjusted for BP group, diabetes, and hyperlipidemia.

All statistical analyses were performed with SPSS version 25.0 statistical software (SPSS Inc., Chicago, IL), and a two-sided *P-*value < 0.05 was considered statistically significant.

## Results

### Demographic Characteristics

A total of 1,081 residents aged 60 years and older were enrolled in this study. There were 500 men (46.3%) and 581 women (53.7%), with a mean age of 67.70 years overall (67.96 years for men and 67.47 years for women). In this rural population, the prevalence of cognitive impairment was 33.2% overall (26.6% in men and 38.9% in women). Moreover, there was a higher prevalence of central and general obesity in women than in men (88.1 vs. 44.0% and 22.5 vs. 16.2%, respectively; both *P* < 0.01) among residents with cognitive impairment. In this population, women were more likely to have lower educational attainment and MMSE scores and higher BMI, DBP, FBG, TC, TG, HDL, and LDL levels than men (*P* < 0.05) (Table 1).

**Table 1:** Demographic characteristics and risk factors for all participants by sex group.

**Risk factors**	**Men**	**Women**	**Total**	***P***
Total, *n* (%)	500 (46.3)	581 (53.7)	1081 (100)	–
Age, means (SD)	67.96 (6.42)	67.47 (6.48)	67.70 (6.46)	0.209
Age group, *n* (%)				0.201
60–64 years	190 (38.0)	249 (42.9)	438 (40.6)	
65–69 years	138 (27.6)	157 (27.0)	295 (27.3)	
70–74 years	82 (16.4)	72 (12.4)	155 (14.2)	
≥75 years	90 (18.0)	103 (17.7)	193 (17.9)	
Education, years, mean (SD)	5.35 (2.72)	2.62 (2.94)	3.88 (3.15)	<0.001
Education, *n* (%)				<0.001
0–5 years	203 (40.6)	451 (77.6)	654 (60.5)	
6–8 years	219 (43.8)	101 (17.4)	320 (29.6)	
≥9 years	78 (15.6)	29 (5.0)	107 (9.9)	
Smoking, *n* (%)				<0.001
No	136 (27.2)	261 (44.9)	397 (36.7)	
Yes	364 (72.8)	320 (55.1)	684 (63.3)	
Drinking, *n* (%)				<0.001
No	280 (56.0)	492 (84.7)	772 (71.4)	
Yes	220 (44.0)	89 (15.3)	309 (28.6)	
Physical exercise, n (%)				0.779
No	429 (85.8)	495 (85.2)	924 (85.5)	
Yes	71 (45.2)	86 (14.8)	157 (14.5)	
Hypertension, n (%)				0.568
No	106 (21.2)	115 (19.8)	221 (20.4)	
Yes	394 (78.8)	466 (80.2)	860 (79.6)	
Diabetes, *n* (%)				0.012
No	432 (86.4)	469 (80.7)	901 (83.3)	
Yes	68 (13.6)	112 (19.3)	180 (16.7)	
Hyperlipidemia, *n* (%)				<0.001
No	258 (51.9)	190 (32.9)	448 (41.7)	
Yes	239 (48.1)	388 (67.1)	627 (58.3)	
BP group, *n* (%)				0.934
Normal	127 (25.4)	144 (24.8)	271 (25.1)	
Stage I HBP	176 (35.2)	199 (34.3)	375 (34.7)	
Stage II HBP	118 (44.5)	147 (25.3)	265 (24.5)	
Stage III HBP	79 (15.8)	91 (15.7)	170 (15.7)	
BMI (kg/m^2^), mean (SD)	24.59 (3.31)	25.21 (3.57)	24.92 (3.46)	0.004
BMI category (kg/m^2^), *n* (%)				0.009
Normal	220 (44.0)	212 (36.5)	432 (40.0)	
Overweight	199 (39.8)	238 (41.0)	437 (40.4)	
Obese	81 (16.2)	131 (22.5)	212 (19.6)	
Waist (cm), mean (SD)	88.58 (9.29)	89.21 (8.68)	88.92 (8.97)	0.250
Central obesity, *n* (%)				<0.001
No	280 (56.0)	69 (11.9)	349 (32.3)	
Yes	220 (44.0)	512 (88.1)	732 (67.7)	
MMSE score, mean (SD)	22.30 (4.54)	19.13 (5.31)	20.60 (5.21)	<0.001
MMSE group, *n* (%)				<0.001
Non-CI	367 (73.4)	355 (61.1)	722 (66.8)	
CI	133 (26.6)	226 (38.9)	359 (33.2)	
SBP, mean (SD)	153.66 (23.06)	154.95 (23.28)	154.35 (23.18)	0.360
DBP, mean (SD)	87.05 (11.53)	85.52 (11.76)	86.23 (11.67)	0.031
FBG, mean (SD)	5.84 (1.18)	6.18 (1.93)	6.02 (1.64)	0.001
TC, mean (SD)	4.56 (0.95)	5.18 (1.07)	4.89 (1.06)	<0.001
TG, mean (SD)	1.41 (0.83)	1.82 (1.29)	1.63 (1.12)	<0.001
HDL, mean (SD)	1.42 (0.44)	1.53 (0.47)	1.48 (0.46)	<0.001
LDL, mean (SD)	2.49 (0.79)	2.85 (0.90)	2.68 (0.87)	<0.001

*SD, standard deviation; BP, blood pressure; BMI, body mass index; MMSE, Mini-Mental state examination; CI, cognitive impairment; SBP, systolic blood pressure; DBP, diastolic blood pressure; FBG, fasting blood glucose; TC, total cholesterol; TG, triglycerides; HDL, high-density lipoprotein cholesterol; LDL, low-density lipoprotein cholesterol*.

### Associations of Cognitive Impairment With Risk Factors by Sex in the Univariate Analysis

For elderly men, cognitive impairment was associated with older age, lower educational attainment, and higher SBP. Moreover, WC and were associated with cognitive impairment in men. The prevalence of cognitive impairment in men with central obesity was lower than that without central obesity (Table 2).

**Table 2:** Association of cognitive impairment with demographic characteristics and risk factors by gender in univariate analysis.

**Characteristics**	**Male**			**Female**		
	**Non-CI**	**CI**	***P***	**Non-CI**	**CI**	***P***
Age, means (SD)	66.89 (5.71)	70.92 (7.31)	<0.001	66.04 (5.36)	69.71 (7.41)	<0.001
Age group, *n* (%)			<0.001			<0.001
60–64 years	158 (83.2)	32 (16.8)		175 (70.3)	74 (29.7)	
65–69 years	107 (77.5)	31 (22.5)		105 (66.9)	52 (33.1)	
70–74 years	54 (65.9)	28 (34.1)		42 (58.3)	30 (41.7)	
≥75 years	48 (53.3)	42 (46.7)		33 (32.0)	70 (68.0)	
Education, years, mean (SD)	5.64 (2.57)	4.55 (2.97)	<0.001	3.43 (2.99)	1.34 (2.36)	<0.001
Education, *n* (%)			<0.001			<0.001
0–5 years	128 (63.1)	75 (36.9)		247 (54.8)	204 (45.2)	
6–8 years	180 (82.2)	39 (17.8)		84 (83.2)	17 (16.8)	
≥9 years	59 (75.6)	19 (24.4)		24 (82.8)	65 (17.2)	
Smoking, *n* (%)			0.121			0.672
No	93 (68.4)	274 (31.6)		157 (60.2)	104 (39.8)	
Yes	274 (75.3)	90 (24.7)		198 (61.9)	122 (38.1)	
Drinking, *n* (%)			0.922			0.536
No	206 (73.6)	74 (26.4)		298 (60.6)	194 (39.4)	
Yes	161 (73.2)	59 (26.8)		57 (64.0)	32 (36.0)	
Physical exercise, *n* (%)			0.584			0.542
No	313 (73.0)	116 (27.0)		305 (61.6)	190 (38.4)	
Yes	54 (76.1)	17 (23.9)		50 (58.1)	36 (41.9)	
Hypertension, *n* (%)			0.125			0.221
No	84 (79.2)	22 (20.8)		76 (66.1)	39 (33.9)	
Yes	283 (71.8)	111 (28.2)		279 (59.9)	187 (40.1)	
Diabetes, *n* (%)			0.081			0.325
No	323 (74.0)	109 (26.0)		282 (60.1)	187 (39.9)	
Yes	44 (74.8)	24 (25.2)		73 (65.2)	39 (34.8)	
Hyperlipidemia, *n* (%)			0.220			0.908
No	195 (75.6)	63 (24.4)		117 (61.6)	73 (38.4)	
Yes	169 (70.7)	70 (29.3)		237 (61.1)	151 (38.9)	
BP group, *n* (%)			0.024			0.007
Normal	97 (77.0)	30 (23.0)		96 (66.7)	48 (33.3)	
Stage I HBP	135 (78.6)	41 (21.4)		129 (64.8)	70 (35.2)	
Stage II HBP	88 (74.4)	30 (25.6)		83 (56.5)	64 (43.5)	
Stage III HBP	47 (60.2)	32 (39.8)		47 (51.6)	44 (48.4)	
BMI (kg/m^2^), mean (SD)	24.88 (3.25)	24.10 (3.43)	0.047	25.18 (3.53)	25.25 (3.63)	0.797
BMI category, *n* (%)			0.064			0.962
Normal	150 (69.0)	70 (31.0)		131 (61.8)	81 (38.2)	
Overweight	154 (79.2)	45 (20.8)		144 (60.5)	94 (39.5)	
Obese	63 (76.7)	18 (23.3)		80 (61.1)	51 (38.9)	
Waist (cm), mean (SD)	89.14 (9.26)	87.04 (9.25)	0.025	89.96 (9.00)	89.61 (8.15)	0.379
Central obesity, *n* (%)			0.006			0.124
No	192 (68.6)	88 (31.4)		48 (69.6)	21 (30.4)	
Yes	175 (79.5)	45 (20.5)		307 (60.0)	205 (40.0)	
SBP, mean (SD)	152.27 (22.40)	157.49 (24.47)	0.025	152.95 (22.13)	158.10 (24.71)	0.009
DBP, mean (SD)	87.14 (10.91)	86.80 (13.12)	0.790	85.38 (11.41)	85.73 (12.31)	0.729
FBG, mean (SD)	5.79 (1.15)	6.01 (1.26)	0.064	6.23 (2.05)	6.11 (1.74)	0.467
TC, mean (SD)	4.55 (0.97)	4.57 (0.92)	0.812	5.15 (1.06)	5.20 (1.06)	0.400
TG, mean (SD)	1.42 (0.83)	1.41 (0.85)	0.790	1.86 (1.45)	1.75 (0.97)	0.346
HDL, mean (SD)	1.41 (0.42)	1.44 (0.49)	0.535	1.51 (0.44)	1.56 (0.50)	0.184
LDL, mean (SD)	2.49 (0.82)	2.48 (0.73)	0.883	2.84 (0.91)	2.87 (0.90)	0.699
MMSE score, mean (SD)	24.32 (2.69)	16.71 (3.87)	<0.001	22.50 (3.20)	13.85 (3.27)	<0.001

*SD, standard deviation; BP: blood pressure; BMI, body mass index; MMSE, Mini-Mental state examination; CI: cognitive impairment; SBP, systolic blood pressure; DBP, diastolic blood pressure; FBG: fasting blood glucose; TC, total cholesterol; TG, triglycerides; HDL, high-density lipoprotein cholesterol; LDL, low-density lipoprotein cholesterol*.

For elderly women, cognitive impairment was associated with older age, lower educational attainment, and higher SBP. However, neither BMI nor WC was associated with cognitive impairment in women (Table 2).

### Associations of BMI and WC With Cognitive Impairment by Sex and Age in the Multivariate Analysis

For elderly men, there was no significant association between BMI and cognitive impairment after adjusting for age, educational level, and BP group. WC was associated with cognitive impairment in elderly men, and each 1-cm increase in WC was associated with a 3% decrease in the prevalence of cognitive impairment after adjusting for age, educational level, and BP group (95% CI 0.95–0.99; *P* = 0.013). In a subgroup analysis, the association remained consistent among men aged 60–64 years and each 1-cm increase in WC was associated with a 5% decrease in the prevalence of cognitive impairment (95% CI 0.91–0.99; *P* = 0.042) (Table 3).

**Table 3:** Association of BMI and waist circumference with cognitive impairment by age groups among elderly men in the multivariate analysis.

**Factors**	**OR (95% CI)**	***P***
BMI		
All male participants^a^	0.95 (0.89, 1.02)	0.129
Age group		
60–64 years^b^	0.90 (0.79, 1.03)	0.125
65–69 years^b^	1.00 (0.88, 1.14)	0.981
70–74 years^b^	0.97 (0.89, 1.13)	0.691
≥75 years^b^	0.95 (0.82, 1.09)	0.459
Waist circumference		
All male participants^a^	0.97 (0.95, 0.99)	0.013
Age group		
60–64 years^b^	0.95 (0.91, 0.99)	0.042
65–69 years^b^	1.00 (0.96, 1.04)	0.942
70–74 years^b^	0.97 (0.92, 1.03)	0.270
≥75 years^b^	0.96 (0.91, 1.02)	0.161

a *Adjusting for age, education and BP group*.

b *Adjusting for education and BP group*.

BMI was associated with the prevalence of cognitive impairment in elderly women in Models 1, 2, and 3, while BMI was not associated with cognitive impairment in any multivariate model in elderly men. Each 1-unit increase in BMI was associated with a 5.9% increase in the prevalence of cognitive impairment in elderly women after adjusting for age, educational level, smoking, drinking, physical exercise, BP group, diabetes, and hyperlipidemia in Model 3 (95% CI 1.003–1.119; *P* = 0.038; Table 4).

**Table 4:** Association of BMI and waist circumference with cognitive impairment by sex, according to demographic characteristics and risk factor groups in the multivariate analysis.

**Factors**	**Model 1 OR (95% CI)**	**Model 2 OR (95% CI)**	**Model 3 OR (95% CI)**
BMI			
Male	0.961 (0.900, 1.025)	0.963 (0.902, 1.028)	0.938 (0.875, 1.005)
Female	1.058 (1.005, 1.115)^*^	1.057 (1.003, 1.113)^*^	1.059 (1.003, 1.119)^*^
Waist circumference			
Male	0.973 (0.951, 0.997)^*^	0.975 (0.952, 0.998)^*^	0.960 (0.935, 0.984)^*^
Female	1.015 (0.995, 1.036)	1.015 (0.995, 1.037)	1.015 (0.994, 1.037)

*Model 1 analysis adjusted by age and education*.

*Model 2 analysis adjusted by age, education, smoking, drinking and physical exercise*.

*Model 3 analysis adjusted age, education, smoking, drinking, physical exercise, BP group, diabetes and hyperlipidemia*.

* *Represents P < 0.05*.

For elderly men, WC was related to the prevalence of cognitive impairment in Models 1, 2, and 3. Each 1-cm increase in WC was associated with a 4.0% decrease in the prevalence of cognitive impairment after adjusting for age, educational level, smoking, drinking, physical exercise, BP group, diabetes, and hyperlipidemia in Model 3 (95% CI 0.935–0.984; *P* = 0.002). However, there was no significant association between WC and cognitive impairment in elderly women (Table 4).

This study further explored the relationships between cognitive impairment with sex-specific quartiles of BMI and WC. Compared with the first quartile of BMI, the OR of the third quartile group among elderly women was 1.79 (95% CI 1.05–3.05) after adjusting for age, education, and BP group. For elderly men, the risk of cognitive impairment in the highest WC quartile decreased by 55% when compared with that in the lowest quartile (Table 5).

**Table 5:** Association of BMI and waist circumference quartiles with cognitive impairment by sex in the multivariate analysis.

**Characteristics**	**References**	**OR (95% CI)**	
		**Male**	**Female**
BMI^a^	First quartile		
Second quartile		0.91 (0.52, 1.57)	0.94 (0.54, 1.63)
Third quartile		0.63 (0.35, 1.14)	1.79 (1.05, 3.05)^*^
Fourth quartile		0.66 (0.35, 1.23)	1.25 (0.73, 2.14)
WC ^a^	First quartile		
Second quartile		0.84 (0.47, 1.48)	1.02 (0.60, 1.75)
Third quartile		0.64 (0.36, 1.16)	1.41 (0.85, 2.32)
Fourth quartile		0.45 (0.24, 0.81)^**^	1.25 (0.73, 2.15)

a *Adjusting for age, education and BP group*.

* *represents P < 0.05;*

** *represents P < 0.01*.

When the interaction term was introduced, the analyses revealed a significant interaction between sex and BMI as well as sex and WC (Table 6).

**Table 6:** The interaction analysis between sex and BMI/ WC in multivariate logistic regression analysis.

**Variables**	**OR**	**95% CI**	***P***
Model 1^a^			
Sex	0.152	0.019, 1.185	0.072
BMI	0.956	0.894, 1.022	0.186
Sex*BMI	1.093	1.006, 1.187	0.035
Model 2^b^			
Sex	0.033	0.002, 0.535	0.016
WC	0.970	0.947, 0.993	0.012
Sex*WC	1.043	1.011, 1.076	0.008

a *Model 1 was adjusted for age, education, blood pressure groups and interaction terms sex by BMI*.

b *Model 2 was adjusted for age, education, blood pressure groups and interaction terms sex by WC*.

## Discussion

The relationship between obesity and cognitive impairment in old age has been a controversial issue for many years. In the present population-based study, we evaluated the sex differences in the associations of BMI and WC with cognitive impairment in a low-income elderly population in rural China. We found that BMI was related to the prevalence of cognitive impairment in elderly women, not in elderly men; each 1-unit increase in BMI corresponded to a 5.9% increase in cognitive impairment in elderly women. Besides, each 1-cm increase in WC corresponded to a 4.0% decrease in prevalence of cognitive impairment in the fully adjusted model in elderly men. In the age subgroup analysis, the association remained consistent among men aged 60–64 years. However, there were no significant associations between WC and cognitive impairment in elderly women.

Obesity is often recognized as a public health issue worldwide because of its contribution to increased mortality and morbidity from cardiovascular diseases (26). However, the association between BMI and cognitive decline in the elderly is complex and controversial. Most studies have reported that being overweight in later life was associated with a lower risk of cognitive impairment and dementia in the elderly, which supported the “jolly fat” hypothesis of cognitive function (18, 20, 27–29). A clinical retrospective cohort study over two decades was conducted in the UK, and almost two million participants aged 40 years and older were included. In that study, being overweight or even obese in middle age and old age was associated with a decreased risk of dementia. Obese individuals (i.e., BMI >40 kg/m^2^) had a 29% lower (95% CI 22–36%) dementia risk than individuals with a healthy weight, while underweight people (i.e., BMI <20 kg/m^2^) had a 34% higher (95% CI 29–38%) risk of dementia (30). However, a recent systematic review failed to find a beneficial effect of being overweight on cognitive function in later life (31). Additionally, a few studies have reported that obesity is negatively associated with cognitive function in old age (16, 17). A population-based cross-sectional study in central Spain suggested that overweight and obese elderly adults had poorer neuropsychological performance than elderly adults with normal weight in verbal fluency, delayed-free recall, and immediate logical memory (16). Another population-based study revealed that increased BMI and visceral fat mass were associated with severe cognitive impairment in older men (17). In our study, a high BMI was associated with an increased prevalence of cognitive impairment in elderly women. In contrast, a high BMI appeared to be a protective factor for elderly men, although not to a significant degree, which was partly consistent with results of previous studies (18, 20). A prospective study of community-dwelling Koreans demonstrated that increased obesity, as defined using BMI, over time was associated with better cognitive function in elderly men. However, for elderly women, increased obesity, as defined using WC and waist-to-hip ratio (WHR), over time was associated with cognitive decline (18). The beneficial effects of obesity seemed to be applicable to elderly men and not to elderly women.

WC is a more accurate indicator of abdominal visceral obesity than BMI, percent body fat, and WHR (32). Moreover, abdominal obesity as classified using WC is a component of metabolic syndrome and is generally regarded as harmful to health. Most previous studies have demonstrated that a large WC is a risk factor for both cognitive impairment and dementia in the elderly (13, 16, 33–35). A longitudinal community-based study with an 8-year follow-up period revealed that abdominal obesity in late life carried an increased risk of cognitive impairment. Compared with the low WC tertile, the middle and high WC tertiles were associated with 80 and 90% higher incidence rates of cognitive impairment, respectively (13). In China, a hospital-based study found that abdominal obesity as defined by WHR was an independent risk factor for cognitive impairment (OR 1.532; 95% CI 1.037–2.263) (36). In contrast, we found that a large WC was associated with a decreased prevalence in cognitive impairment in elderly men, independent of sociodemographic, lifestyle, and health-related factors. Each 1-cm increase in WC corresponded to 4.0% decrease in prevalence of cognitive impairment in elderly men. Furthermore, results of two Asian population-based studies are consistent with our findings. One population-based study suggested that abdominal obesity (i.e., WC ≥90 cm for men and ≥80 cm for women) might be a protective factor for cognitive function in the elderly (OR 0.72; 95% CI 0.56–0.92) in the rural area of Shenyang, China (37). In addition, a prospective community-dwelling Korean study illustrated that WC had a U-shaped correlation with cognitive function in the elderly, and increased central obesity over time as assessed using WC and WHR was associated with better cognitive function in elderly men (18).

In addition to obesity indicators, other cardiovascular risk factors, including hypertension, diabetes mellitus, hyperlipidemia, and smoking, have been linked with cognitive decline in adults (38–40). In our previous studies, a high blood pressure proved to be a risk factor for cognitive impairment (9, 19). However, there was no association between diabetes and cognitive impairment in this population (9, 19). Previous studies have reported that hyperlipidemia is associated with cognitive impairment (41, 42). Several studies suggested both WC and BMI were ideal indicators for hyperlipidemia (43, 44). A cross-sectional study found BMI and WC were comparable to body fat mass in identifying metabolic risk factors including hyperlipidemia (45). In present study, after adjusting for hyperlipidemia and other confounding factors, BMI and WC were associated with cognitive decline in elderly women and men, respectively.

In this study, there were different associations between obesity and cognitive function according to sex. Obesity appears to be protective for cognitive function only in elderly men, not elderly women. Several mechanisms may account for these results. First, female sex is a risk factor for cognitive decline (9). Women are more likely to develop central obesity in this population. Second, testosterone is an important neuroprotective hormone, as it decreases the accumulation of amyloid β-protein and inhibits the hyperphosphorylation of tau (46). Men are likely to maintain more testosterone in body fat, which may prevent cognitive decline (47). Third, in rural China, men tend to obtain higher educational levels, have more social contact, and be less depressed. Therefore, they are more likely to seek cognitive rehabilitation to improve cognitive disorders.

There are several limitations in this study. First, the study population was from a low-income, low-education rural population in northern China; thus, its representation and generalizability are limited. Second, cognitive impairment was evaluated with MMSE scores; therefore, different types of cognitive impairment could not be further diagnosed in the study. Third, other confounding factors, including apolipoprotein E genotype, frailty, and diet, are important factors for cognitive decline but were not assessed in this study because of limited funding. In the future, we plan to conduct a study including physical capability markers. Fourth, this was a cross-sectional study; thus, causal relationships could not be established. A prospective cohort study will be conducted using this study as reference.

## Conclusion

A higher WC was positively associated with better cognitive function in low-income elderly men in rural China, whereas a higher BMI was associated with increased cognitive impairment in low-income elderly women in China, independent of sociodemographic, lifestyle, and health-related comorbid factors. The beneficial effects of obesity on cognitive impairment seemed to be applicable to elderly men, not women. Our results suggest weight management of elderly women in rural China may have cognitive benefits. However, randomized controlled trials would be needed to confirm causality.

## Data Availability Statement

The raw data supporting the conclusions of this article will be made available by the authors, without undue reservation.

## Ethics Statement

The studies involving human participants were reviewed and approved by the Ethics Committee of Tianjin Medical University General Hospital. The patients/participants provided their written informed consent to participate in this study.

## Author Contributions

JW, XN, and YS contributed to the study design, performed data collection, data interpretation, and critical review. JW performed data analysis. DG, XZ, and CZ contributed to drafting of the article. DG, XZ, CZ, QL, JL, QY, and JT performed data collection, case diagnoses, and confirmation of case diagnoses. All authors read, revised, and approved the final version of the paper.

## Conflict of Interest

The authors declare that the research was conducted in the absence of any commercial or financial relationships that could be construed as a potential conflict of interest.
